# Two-Ply Composite Membranes with Separation Layers from Chitosan and Sulfoethylcellulose on a Microporous Support Based on Poly(diphenylsulfone-*N*-phenylphthalimide)

**DOI:** 10.3390/molecules22122227

**Published:** 2017-12-14

**Authors:** Svetlana V. Kononova, Elena V. Kruchinina, Valentina A. Petrova, Yulia G. Baklagina, Kira A. Romashkova, Anton S. Orekhov, Vera V. Klechkovskaya, Yury A. Skorik

**Affiliations:** 1 Institute of Macromolecular Compounds of the Russian Academy of Sciences, Bolshoi pr. VO 31, St. Petersburg 199004, Russia; svkononova@list.ru (S.V.K.); evkruchinina@mail.ru (E.V.K.); valentina_petrova_49@mail.ru (V.A.P.); ygbaklagina@mail.ru (Y.G.B.); 1482829@mail.ru (K.A.R.); 2 Shubnikov Institute of Crystallography, Federal Scientific Research Centre “Crystallography and Photonics”, Russian Academy of Sciences, Leninskiy pr. 59, Moscow 119333, Russia; orekhov.anton@gmail.com (A.S.O.); klechvv@crys.ras.ru (V.V.K.); 3 National Research Centre “Kurchatov Institute”, Akademika Kurchatova pl. 1, Moscow 123182, Russia; 4 Institute of Experimental Medicine, Almazov National Medical Research Centre, Akkuratova ul. 2, St. Petersburg 197341, Russia

**Keywords:** two-ply membrane, chitosan, sulfoethylcellulose, polyelectrolyte complex, poly(diphenylsulfone-*N*-phenylphthalimide), porous substrate, pervaporation

## Abstract

Two-ply composite membranes with separation layers from chitosan and sulfoethylcellulose were developed on a microporous support based on poly(diphenylsulfone-*N*-phenylphthalimide) and investigated by use of X-ray diffraction and scanning electron microscopy methods. The pervaporation properties of the membranes were studied for the separation of aqueous alcohol (ethanol, propan-2-ol) mixtures of different compositions. When the mixtures to be separated consist of less than 15 wt % water in propan-2-ol, the membranes composed of polyelectrolytes with the same molar fraction of ionogenic groups (-NH_3_^+^ for chitosan and -SO_3_^−^ for sulfoethylcellulose) show high permselectivity (the water content in the permeate was 100%). Factors affecting the structure of a non-porous layer of the polyelectrolyte complex formed on the substrate surface and the contribution of that complex to changes in the transport properties of membranes are discussed. The results indicate significant prospects for the use of chitosan and sulfoethylcellulose for the formation of highly selective pervaporation membranes.

## 1. Introduction

Increasing attention is now being paid to the formation of interpolymer complexes. One useful component for the creation of these complexes is the relatively rigid-chain chitosan (CS), which has ionogenic groups that allow the formation of intermolecular ion-ion and ion-dipole bonds. This means that CS can improve the mechanical properties of the resulting films, thereby increasing their potential possibilities for different applications. The interaction of oppositely charged polyelectrolyte molecules results in the formation of polyelectrolyte complexes (PECs) that show high hydrophilicity. This feature allows PECs to serve as effective flocculants and structurants, while their films function as semipermeable membranes and coatings, including those used in medicine. PEC films have also been successfully used in the separation of water-organic mixtures by pervaporation (PV) [1].

PV membranes composed of diffusion separation layers based on PEC can show significant differences in their structural and morphological characteristics. No unambiguous and accurate classification of PEC membranes is yet available, but several structural types are typically distinguished. The PEC layers of these membranes can be formed in several ways, including counterion mixing [2], polymer network preparation [3], and interfacial complexation. The main difference between solution mixing and interfacial complexation methods is that the interfacial method involves fixation of one component polyelectrolyte onto a solid surface. The choice of method for PEC layer formation on the surface of a porous substrate can affect the transport properties of the resulting membrane. The deposition from a solution of polymer electrolytes and the layered deposition both produce material types that are quite effective for the PV separation of water-organic mixtures [4].

Two-ply casting or layer-by-layer assembly methods can also be used to deposit a PEC layer from polyelectrolyte solutions onto a porous substrate. These methods can reduce the thickness of the effective PEC layer, while significantly improving the mechanical strength of the membrane. The formation of multilayer membranes of this type has been described in the literature [5,6]; however, a significantly large number of layer-by-layer depositions of anionic/cationic polyelectrolytes are often required to achieve high membrane selectivity. This is especially the case when the substrate membrane is microporous [7], as the presence of open pores on the membrane surface creates membrane defects that give rise to non-selectivity. From a manufacturing point of view, the time required for formation of this type of membrane is also too long to be practical, particularly when a large number of deposition cycles are required. Therefore, commercial applications require procedures that involve fewer polyelectrolyte depositions but still produce defect-free membranes with good permselectivity [7,8]. Consequently, simpler two-ply membranes are now viewed as more promising for commercial applications, provided that the resulting multilayered composite retains high efficiency for the targeted separation.

Some studies have reported the preparation and characterization of two-layer membranes with separation layers in the form of PECs based on CS. The best-known membranes of this type contain alginates as the polyanionic component [9,10,11]. The literature also contains accounts of effective PV separation of water-organic mixtures by PEC membranes containing CS in combination with other polymer counterions, including poly(meth)acrylic acid [12], sodium carboxymethylcellulose [13], carboxymethylchitosan [14,15], carrageenan [16], and other polymers. However, in most cases, the resulting CS-polyanion membranes are not two-ply PEC composite membranes, because the formation of a defect-free, non-porous, and selective PEC layer is quite difficult. In the absence of a planar PEC orientation, the formation of a PEC can induce a conformational change in the other polyelectrolyte, if the latter has a non-rigid structure (e.g., polyacrylic acid, xylan, or collagen). This is due to the rigid, stereo-regular structure of CS [17]. A CS-polyanion film formed on the surface of a microporous substrate can therefore show substantial structural variations.

Our previous work [18,19] investigated the complexation process occurring between CS and the sulfoethylcellulose (SEC) in the preparation of multilayer self-standing PEC films. We first established the film structure, especially that of the PEC layer, using X-ray diffraction (XRD), scanning electron microscopy (SEM), and energy-dispersive X-ray microanalysis. We found that the interaction between the positively charged groups of CS (i.e., -NH_3_^+^) and the negatively charged groups of SEC (i.e., -SO_3_^−^) in the multilayer film achieved the packing of the CS chains. Subsequent XRD analysis indicated that the formation of PEC between the CS and SEC layers was accompanied by conformational changes that led to the crystallization of the CS chains.

Assumptions were made previously regarding the promising prospects for the creation of two-ply PV membranes with CS-SEC separation layers [20], but the literature contains almost no concrete data. One reason for this is the difficulty of obtaining mechanically strong and defect-free films from CS and SEC. A second reason is that, even in the case when the preparation of this type of film is possible, the membrane can quickly be destroyed during the PV process because of uneven swelling due to the presence of water in the feed mixtures. The known attempts to obtain CS-SEC membranes, and the studies of their PV properties have indicated the necessity of using a microporous support for a two-ply composite structure. In the absence of a supporting membrane layer, the composite membrane has too low a mechanical strength for practical applications [21,22]. The method of preparation of composite diffusion membranes containing non-porous polymer layers on a porous polymer substrate is commonly used to increase the permeability of the membrane by reducing the thickness of the separation layer [23]. Layered composite membranes have also been prepared to exploit the separating abilities of the substrate material [24]. We used this technique in earlier work describing the preparation of pervaporation membranes supported by a microporous film of aromatic poly(amide imides) [25,26,27]. The substrate can significantly affect both the mechanical and transport properties of the membranes. This depends in particular on the structural and morphological features at the interface of the substrate and the diffusion layer, and the formation of layers on the surface of the substrate can lead to changes in structure and morphology in the diffusion layer itself [25,26,27].

The present work represents an attempt to form multilayered two-ply composite membranes containing separating PEC layers derived from CS with a degree of deacetylation (DDA) of 0.80 (CS_0.8_) and SEC with different degrees of substitution (DS) of 0.40, 0.80 and 1.0 (SEC_0.4_, SEC_0.8_, and SEC_1.0_, respectively). As a microporous support for successive deposition of polyelectrolyte layers was chosen an asymmetric microporous membrane of poly(diphenylsulfone-*N*-phenylphthalimide) (PAI-SO_2_). The information available on the structural and morphological characteristics of this substrate suggests the possibility of forming defect-free PEC layers on its working surface. Thus, the purpose of the present study was to obtain a series of novel two-ply CS-SEC membranes supported by a porous PAI-SO_2_ film and to investigate their PV properties in connection with their structural and morphological features.

## 2. Results and Discussion

### 2.1. Preparation of CS-SEC Membranes on PAI-SO_2_ Support

Two-ply membranes were produced by first forming an asymmetric microporous support. One important requirement was the use of well-characterized support with a smooth surface on the top layer. Therefore, the choice of support was a porous PAI-SO_2_ membrane formed under the conditions described in [28]. The size of the voids in the cross-section of this support decreases in the direction towards the top surface, where in a surface region the so named skin layer is formed. The morphology of the PAI-SO_2_ support was previously described, and the pores in the skin layer of the asymmetric membrane were visualized [28,29]. The concentration of pores in the skin layer did not change significantly in the different samples and was about (3–5) × 10^9^ cm^−2^. The diameter of the skin layer pores of the membrane ranged from 2 to 10 nm, with an average value of about 3 nm. The detailed morphology of the skin layer (working surface) was assumed to contribute to the formation of a defect-free coating. In other words, the first non-porous polyelectrolyte layer formed on the support surface must be continuous and defect-free. The casting of a smooth, defect-free layer of polyelectrolyte that contacts the support creates a clear boundary between the layers of polyelectrolytes and facilitates the formation of the PEC.

CS is known to dissolve easily at low pH, whereas it is insoluble at higher pH due to pH-sensitive swelling. The underlying mechanism involves the protonation of the CS amine groups at low pH. This protonation leads to chain repulsion, the diffusion of protons and counter ions together with water inside the gel, and the occurrence of secondary interactions [3]. The DDA determines the solubility of CS in acidic solution, as well as its ability to re-form supramolecular structures via hydrogen bonding after evaporation of the solvent. The CS swellability rises with its increasing molar mass and decreasing DDA [3,30]. Therefore, we used only one sample of CS with a molecular weight (MW) of 1.7 × 10^5^ and a DDA of 0.8 for all experiments.

The need to clarify the contribution of the PAI-SO_2_ support to the transport properties of the membranes, not only because of its flow resistance, but also the possible effect on the structural and morphological shape of the CS-SEC polyelectrolyte layers formed on its surface, made it necessary to obtain these layers under the same conditions under which self-supporting CS-SEC films were formed. For this reason, the layers of polyelectrolytes were deposited on a substrate from 3 wt % solutions. Notably, little success was achieved in forming diffusion CS-SEC layers of a smaller thickness on the surface of the PAI-SO_2_ support with the use of lower concentrations of polyelectrolyte solutions (2% or less). The obtained samples had microdefects of their structure, did not withstand tests in the pervaporation cell, and they could not be used for the study.

### 2.2. Scanning Electron Microscopy Morphology Characterization

**Figure 1:**
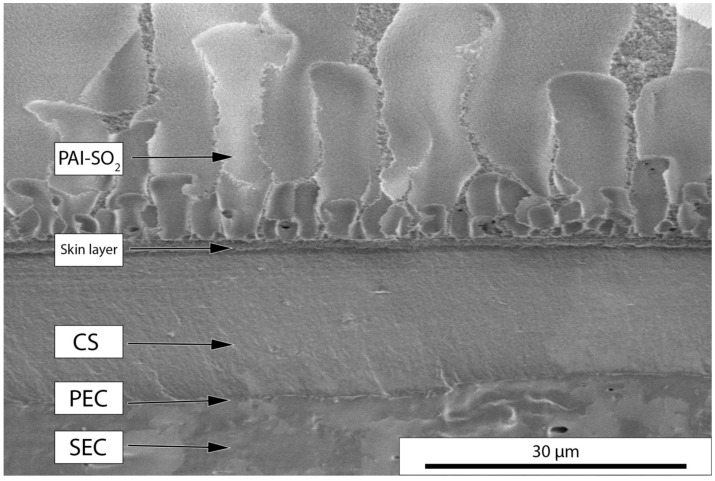
Low voltage scanning electron microscopy (LVSEM) images of the cross-section of SEC_0.8_-PEC-CS_0.8_/PAI-SO_2_ membrane (SEC_0.8_, sulfoethylcellulose with a degree of substitution of 0.80; PEC, polyelectrolyte complex; CS_0.8_, chitosan with a degree of deacetylation of 0.80; PAI-SO_2_, poly(diphenylsulfone-*N*-phenylphthalimide).

The low voltage scanning electron microscopy (LVSEM) images of the cross-section of the multilayer membrane were recorded at a voltage of 1 kV and 86 pA current, as described in detail previously [31]. To test a new technique a multilayer polymer film containing non-porous composite layers was prepared. The LVSEM images of this sample (Figure 1) demonstrated a clear boundary between the support surface PAI-SO_2_ (with skin layer of 1.1 μm in thickness) and the diffusion layer of the resulting two-ply composite membrane (contains CS layer of 14.5 μm in thickness, and SEC_0.8_ layer of 16 μm in thickness). The boundary between the layers of polymer counterions was also visible in the micrographs. The very thin layer present at this boundary was identified as PEC [18,19].

A clear line was evident between the polymer counterions in the cases where SEC_0.8_ was used, and in any deposition sequence for the layers (i.e., SEC_0.8_-PEC_0.8_-CS_0.8_/PAI-SO_2_ or CS_0.8_-PEC_0.8_-SEC_0.8_/PAI-SO_2_). The interphase layer between SEC and CS was first visualized on a model CS_0.8_-PEC_0.8_-SEC_0.8_ film in our previous work [18]. In the present study, our aim was to determine how clear a boundary region is realized between SEC and CS during the formation of the layers on the PAI-SO_2_ support.

**Figure 2:**
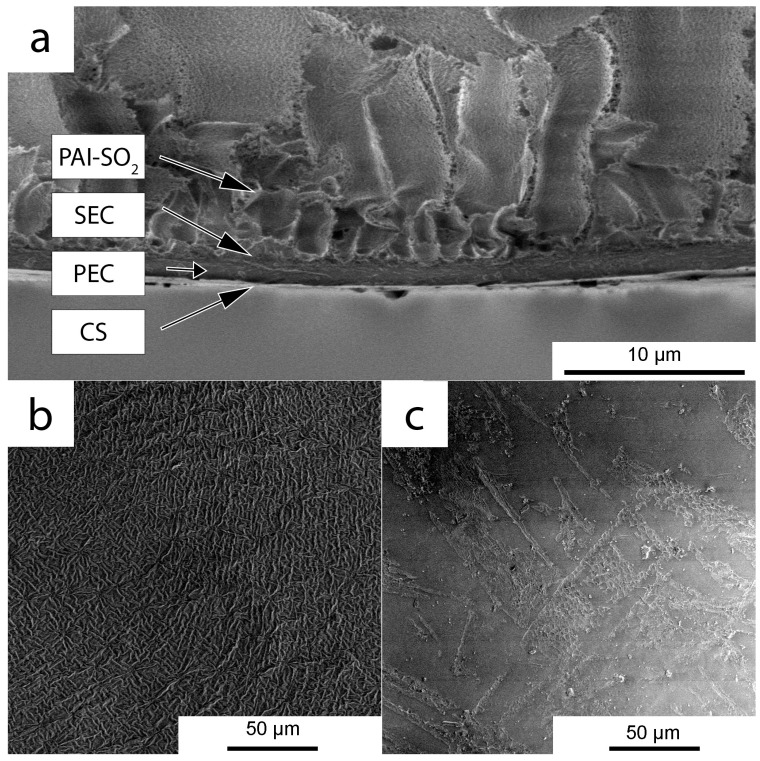
LVSEM images of the cross-section of CS_0.8_-PEC-SEC_0.4_/PAI-SO_2_ (**a**); and the surface after water washing to remove the SEC from the SEC_0.8_-PEC-CS_0.8_ (**b**) and SEC_0.4_-PEC-CS_0.8_ (**c**) films.

Figure 2a shows that, for the CS_0.8_-PEC-SEC_0.4_/PAI-SO_2_ membrane, a clear boundary is present between the layers of CS and SEC, but the intermediate layer of the PEC that appears in the cross section is not continuous. This is confirmed by examination of the SEM images of the surface after washing off the SEC layer (Figure 2b,c) from the SEC_0.8_-PEC-CS_0.8_ and SEC_0.4_-PEC-CS_0.8_ model films. The PEC layer is characterized by a tightly packed domain structure with domain size up to 5 μm, as observed on the SEM image of the surface after washing the SEC_0.8_ with water (Figure 2a). When using SEC_0.4_, this dense PEC structure is absent from the washed surface (Figure 2b). The PEC domains are located on the smooth surface of the CS layer, but this layer is not continuous.

### 2.3. X-ray Diffraction

**Figure 3:**
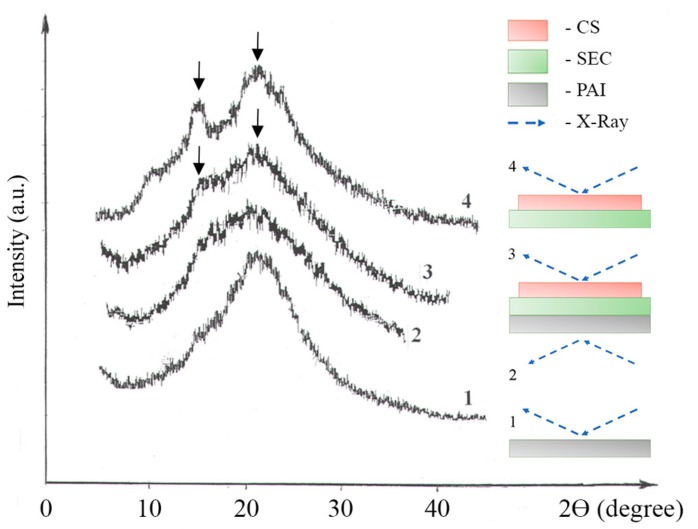
X-ray diffraction (XRD) patterns obtained by reflection from membrane samples: (1) microporous support; (2) the CS_0.8_-PEC-SEC_0.8_/PAI-SO_2_ membrane, as viewed from the support side; (3) the CS_0.8_-PEC-SEC_0.8_/PAI-SO_2_ membrane, as viewed from the CS side; (4) the CS_0.8_-PEC-SEC_0.8_ film, as viewed from the CS side.

The structure of the membrane was studied using a multilayer CS_0.8_-PEC-SEC_0.8_/PAI-SO_2_ film and compared with the model film CS_0.8_-PEC-SEC_0.8_. Figure 3 shows the X-ray diffraction patterns obtained by the reflection from the membrane sample formed as a result of layer-wise deposition of the SEC_0.8_ solution onto the microporous support, followed by deposition of CS_0.8_ solution onto the SEC_0.8_/PAI-SO_2_ composite film. (The ratio of the number of positively charged -NH_3_^+^ groups in CS and negatively charged -SO_3_^−^ groups in SEC was 1:1).

Analysis of these data showed that the formation of the PEC layer by the deposition of CS and SEC layers on the PAI-SO_2_ support was characterized by crystallization of the CS, as previously found for the model CS_0.8_-PEC-SEC_0.8_ film (Figure 3, curve 4). The previous study showed an unexpected result during the search for a specific structure of the PEС layer in model films of the CS-PEC-SEC type [18,19]. The films showed crystallization of CS in the anhydrous allotropic modification, initiated by the formation of a PEC between the polycation and polyanion layers. The ratio of ionogenic groups in the CS and SEC (DDA/DS ratio) determined the properties of the PEC and the degree of CS transformation into the anhydrous polymorph. This crystallization was evident by the appearance of reflections at around 2θ of 15° and 20° on the XRD (Figure 3, curve 3).

### 2.4. Pervaporation

Table 1 shows the pervaporation properties of two-ply membranes, their diffusion layers were formed on the surface of the substrate as results of successive coating counter ion layers. All membranes presented in the Table 1 were formed under the same conditions and differ by (1) the casting sequence of the polyelectrolyte layers during the formation of the diffusion layer; (2) the number of ionogenic groups in the used samples of SEC (different DS). Despite these differences in composition and structure, the membranes seemed to have close layer thicknesses and, consequently, similar flux values for propan-2-ol (entries # 1-4, 4-3, 6-2, Table 1).

As indicated by the data presented in the Table 1, when the feed mixtures contain more than 20 wt % water in ethanol, none of the defect-free membranes obtained are selective (entries # 1-5, 2-1, 3-1, 4-1, 5-1, 6-3, Table 1). Moreover, they show low selectivity when the water/ethanol mixtures contain ≤20 wt % water. Note that membranes with diffusion layers composed of polymer counterions containing approximately the same number of ionogenic groups are the least selective when separating water/ethanol mixtures (entries # 1-6 and 2-2, Table 1). The values of the total fluxes are low in this case, which indicates the absence of any large defects in the diffusion layers of the membranes. At the same time, the CS_0.8_-PEC-SEC_0.8_/PAI-SO_2_ membrane, whose CS layer is in contact with the feed solution in the pervaporation cell, has a low permeability that prevents an accurate measurement of the PV properties during the separation of mixtures of water/propan-2-ol (data are not presented). By contrast, the SEC_0.8_-PEC-CS_0.8_/PAI-SO_2_ membrane is more permeable and highly selective for the separation of aqueous propan-2-ol mixtures containing less than 20 wt % water. In fact, when the feed mixtures contain 15 wt % water or less, the membrane passes chromatographically pure water (entry # 1-3, Table 1).

This result can be explained as follows: in both cases, CS crystallizes in the PEC layer, but in the multilayer CS_0.8_-PEC-SEC_0.8_/PAI-SO_2_ films, the membrane structure has fewer defects, which is manifested as the low permeability of the membrane. However, the PEC layer contains defects in the structure and micro-regions of the amorphous phase. The result is that the membrane is not selective for the separation of ethanol-water mixtures, but it apparently has a high permselectivity for propan-2-ol-containing mixtures at very low permeability. Therefore, we can assume this high selectivity, but we cannot detect it. Another membrane, the CS_0.8_-PEC-SEC_0.8_/PAI-SO_2_, shows a very high selectivity. Since the SEC layer is more hydrophilic (i.e., it contacts the separated mixture), the flow of liquid through the membrane is much larger than occurs in the case of the SEC_0.8_-PEC-CS_0.8_/PAI-SO_2_ membrane. Thus, SEC_0.8_-PEC-CS_0.8_/PAI-SO_2_ membranes (entries # 1-2 and 1-3, Table 1) are distinguished by high permselectivity, and entry # 1-2 also has a significant permeability.

**Figure 4:**
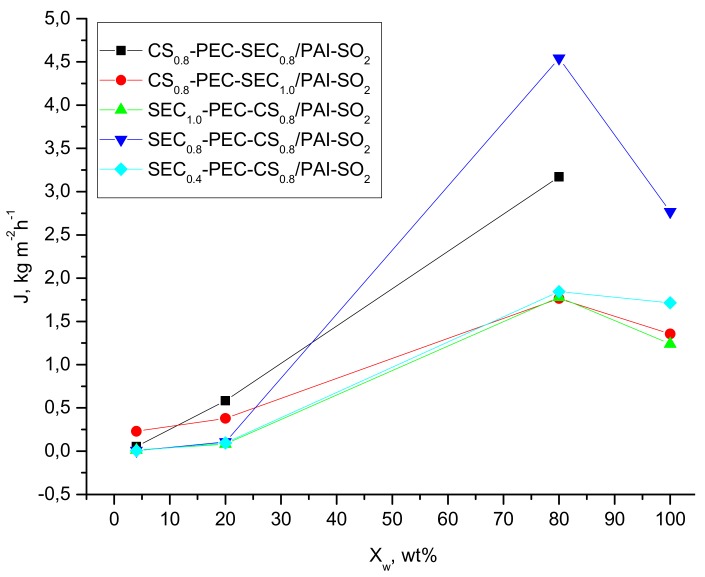
Pervaporation properties at 40 °C for two-ply composite films for water/ethanol mixtures: flux vs. water concentration in the feed.

Membranes containing SEC with different DSs were also investigated in more detail. For all samples, the permeability of the membrane increases with increasing water concentration in the feed mixture, as shown in Figure 4 and in Table 1. A decrease in the concentration of water in the feed increases the membrane permselectivity slightly, but for a feed with 70–80 wt % ethanol, the separation factor exceeds 180 (entries # 5-2 and 6-4, Table 1), which corresponds to 98 wt % water in the permeate. For SEC_0.4_-PEC-CS_0.8_/PAI-SO_2_ over a wide feed range, the water concentration in the permeate is 82 wt %, despite the large differences in permeate fluxes.

Analysis of the data in Table 1 reveals that the fluxes of liquids through a membrane do not depend on the DS of the SEC used, but they do depend on the ordering of the layers. When water is separated from 96% ethanol solutions, membranes having a top layer of CS have low selectivity (*S_F_^w^* = 13) and moderate permeability (entry # 6-5; Table 1). By contrast, for membranes having a SEC in the top layer, the separation factor is larger by an order of magnitude (*S_F_^w^* = 108 for SEC_0.4_-PEC-CS_0.8_/PAI-SO_2_; *S_F_^w^* = 150 for SEC_1.0_-PEC-CS_0.8_/PAI-SO_2_), while the flux through the membrane is reduced by two orders of magnitude (entries # 3-2 and 5-3; Table 1). This finding is particularly interesting because, in this case, a more hydrophilic and amorphous SEC layer is in contact with the feed. Apparently, at such a low concentration of water in the separated mixture, the structure of the PEC layer becomes the key factor that influences the PV properties.

## 3. Materials and Methods

### 3.1. Materials

The crab chitosan (Bioprogress, Shchyolkovo, Russia) used in this study had a MW of 1.7 × 10^5^ (determined by viscometry) and a DDA of 0.8 (determined by conductometric titration).

Sulfoethylcellulose samples with MW of 9000, 14,000, and 40,000 (determined by viscometry) with a degree of substitution of 1.0 (SEC_1.0_), 0.80 (SEC_0.8_), and 0.40 (SEC_0.4_), respectively, were prepared according to a published procedure [32] using the reaction of cellulose with sodium 2-chloroethanesulfonate (Sigma-Aldrich, Budapest, Hungary). The DS was calculated using the elemental (S and C) analysis data.

**Figure 5:**
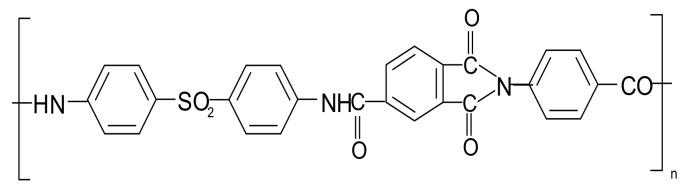
Poly(diphenylsulfone-*N*-phenylphthalimide).

Poly(diphenylsulfone-*N*-phenylphthalimide) (PAI-SO_2_, Figure 5) was synthesized by low-temperature condensation polymerization [33]. The reaction was carried out in *N*-methylpyrrolidin-2-one (NMP, Sigma-Aldrich, Budapest, Hungary), first at −15 °С for 1 h and then at room temperature for 2 h. The synthesis conditions were selected so that the resulting polymer samples had a reduced viscosity of 2.0–2.2 dL·g^−1^ of their 0.5 wt % solutions in NMP.

### 3.2. Membrane Preparation

The asymmetric micro-porous supporting film (membrane support) was prepared from PAI-SO_2_ as a phase-inversion membrane using a previously described technique [28,29]. Support samples were prepared by a dry-wet method that involved immersing gel-films of PAI-SO_2_ in NMP on glass plates into an aqueous precipitation bath.

Two-ply composite membranes were formed by the following methods:

- Coating 3 wt % aqueous solutions of SEC_0.4_, SEC_0.8_, or SEC_1.0_ onto the surface of the top layer (skin layer) of a microporous PAI-SO_2_ support and removing the excess solution after its minimal contact with the surface to obtain intermediate composites. After drying these composites (SEC_0.4_, SEC_0.8_, or SEC_1.0_/PAI-SO_2_) in air, the 3 wt % CS solution in aqueous acetic acid was cast onto the surface of the SEC layer, followed by removal the solvent in ambient conditions and then by vacuum degassing at 30 °C.
- Coating the 3 wt % CS solution in aqueous acetic acid onto the surface of the skin layer of the PAI-SO_2_ substrate to obtain the intermediate composite CS_0.8_/PAI-SO_2_, followed by casting the SEC-layer onto the surface of the CS layer and drying as already described.

These casting conditions were chosen to obtain a defect-free separation layer, which was confirmed by the stability of the residual pressure in the PV cell. As a result, a two-ply composite membrane consists of a non-porous film derived from CS and SEC (separation layer) in contact with the asymmetric microporous PAI-SO_2_ film (support).

Using this method, the following membrane structures were prepared: CS_0.8_-PEC-SEC*_x_*/PAI-SO_2_ and SEC*_x_*-PEC-CS_0.8_/PAI-SO_2_ (*x* = 0.4, 0.8, or 1.0). This designation allows the focus of attention to be on the PEC layer inside the non-porous separation layer of the two-ply composite membrane.

The model non-porous SEC_0.8_-PEC-CS_0.8_ and SEC_0.4_-PEC-CS_0.8_ films were prepared as described in our previous work [18]: the deposition of polyion solution (3% CS solution in 2% solution of acetic acid) on a balanced glass substrate, drying it to the gel-like state, and subsequently depositing a counter ion solution (3% aqueous solution of SEC) and drying the film at room temperature. The resulting complex films were three-layer systems and contained CS, SEC, and an insoluble PEC layer formed at the interface between two polyion phases.

The PEC-CS_0.8_ films were obtained after treatment of the above films with an ethanolic solution of ammonia (which rendered the CS insoluble in water), followed by extraction of the free SEC with water.

### 3.3. General Methods

The viscosity of the SEC and CS solutions (6% NaOH for SEC and 0.3 М NaCl/2% acetic acid for CS) was measured at 25 °C in a modified Ubbelohde viscometer (Design Bureau Pushchino, Pushchino, Russia) with a capillary diameter of 0.3 mm; the time accuracy was within ±0.1 s. The intrinsic viscosity of the samples was calculated by the extrapolation of the dependence ln(η) × *C*^−1^ to an infinite dilution using the least squares method. The MWs of CS and SEC were calculated using the Mark–Houwink equation: [η] = K × *M*^α^, where [η] is the intrinsic viscosity and K and α are empirical constants [32,34].

The DDA of CS was determined by back alkalimetric titration (0.5 M NaOH) with conductometric detection using a Hanna EC215 conductometer (Hanna Instruments, Laval, Canada). Elemental analysis was performed using a PE 2410 Series II Elemental Analyzer (Perkin Elmer, Waltham, MA, USA).

The XRD studies of the asymmetric microporous PAI-SO_2_ film, the two-ply composite CS_0.8_-PEC-SEC_0.8_/PAI-SO_2_ membrane, and the model SEC_0.8_-PEC-CS_0.8_ film were carried out in reflection geometry using a DRON-3M instrument (Ni-filtered CuK_α_ radiation, λ = 1.5418 Å, 40 kV, 30 mA, Burevestnik, St. Petersburg, Russia).

The microstructure of the surfaces of the composite polymer films and the morphology of their cross-sections were investigated using a Versa 3D (FEI, Hillsboro, OR, USA) scanning electron microscope with a field emission electron source (FEG). Samples were observed at an accelerating voltage of 1 kV to remove the “charge effect”.

### 3.4. Pervaporation Measurements

The PV experiments were performed at feed temperatures of 40 or 51 °C to separate aqueous alcohol (ethanol or propan-2-ol) mixtures of different compositions (see Table 1) with the following measurement technique: vacuum degassing of the space under the membrane on a laboratory autonomous non-continuous flow set-up with an operating membrane area of 1.38 × 10^−3^ m^2^ [28].

Permeate vapors were condensed in a receiver with liquid nitrogen and then weighed. The flux value *J* (kg·m^−2^·h^−1^) was calculated using the following equation:

$$J=\;m\cdot S^{-2}\cdot t^{-1},$$

where *m* is the mass of the substance that permeated through membrane area *S* in time *t*.

The compositions of feed and permeate mixtures were analyzed on a GS-2014 Shimadzu gas chromatograph (Shimadzu, Kyoto, Japan) equipped with a packed Porapak Q GC column (Supelco, Bellefonte, PA, USA, (L = 3.0 m) × (O.D. = 1/8 in.) × (I.D. = 2.1 mm)).

The water/alcohol separation factor for a binary water-alcohol mixture (*S_F_^w^*) was calculated using the equation:

$$S_{F}^{w}=\frac{Y_{w}/Y_{alc}}{X_{w}/X_{alc}},$$

where *Х_w_* and *Y_w_* (wt %) are the concentrations of the more permeable component (water) in the feed and permeate, respectively; *Х_alc_* and *Y_alc_* (wt %) are the concentrations of the less permeable component (alcohol) in the feed and permeate, respectively.

## 4. Conclusions

The analysis of the pervaporation data shows that the CS-SEC membranes are highly selective for the separation of propan-2-ol/water mixtures (up to 100 wt % water in the permeate) but are less selective for the separation of water/ethanol mixtures (up to 98 wt % water in the permeate). At low water concentrations (up to 20 wt %) in a separated water/ethanol mixture, the membranes with an upper chitosan layer (CS-PEC-SEC/PAI-SO_2_) show greater permeability. When separating mixtures with higher water content, the SEC-PEC-CS/PAI-SO_2_ membranes are more permeable. This effect is assumed to reflect the ability of the SEC layer to swell in water. At low water concentrations in the feed mixture, the selective transport of penetrants through the composite membrane depends mainly on the membrane structure.

The formation of two-ply composite membranes using deposition of CS and SEC layers on the surface of the skin layer of a microporous PAI-SO_2_ support results in a non-porous PEC containing a separation layer of complex structure. As previously observed in a model non-porous SEC-PEC-CS film [18], a structuring of the CS occurs in the membrane so that a clear boundary is formed between the CS and SEC layers in which the PEC is formed. The continuity of the PEC layer depends on the degree of crosslinking between polyelectrolytes, which, in turn, depends on the concentration of ionogenic groups. Thus, the use of examples of multilayered membranes of complex morphology composed of non-porous SEC-PEC-CS layers on the surface of a microporous PAI-SO_2_ support (two-ply membrane) confirmed the necessity of accounting for the influence of the PEC layer structure on the membrane transport properties.

## Figures and Tables

**Table 1:** Pervaporation properties of SEC*_x_*-PEC-CS_0.8_/PAI-SO_2_ and CS_0.8_-PEC-SEC*_x_*/PAI-SO_2_ multilayer films (*x* = 0.4, 0.8, 1.0) for the separation of two-component liquid mixtures of water/propan-2-ol or water/ethanol.

Entry #	Film Type	Concentration in Feed (*X*) (wt %)			Concentration in Permeate (*Y*) (wt %)			*T* (°C)	*S_F_^w^*	*J* (kg·m^−2^·h^−1^)
		Propan-2-ol	Ethanol	Water	Propan-2-ol	Ethanol	Water			
1-1	SEC_0.8_-PEC-CS_0.8_/PAI-SO_2_	20	-	80	20	-	80	40	1	3.9
1-2		80	-	20	1	-	99	40	308	0.9
1-3		>85	-	<15	0	-	100	40	>550,000	<0.3
1-4		100	-	-	100	-	-	40	-	0.01
1-5		-	20	80	-	21	79	40	1	4.5
1-6		-	80	20	-	35	65	40	7	0.1
2-1	CS_0.8_-PEC-SEC_0.8_/PAI-SO_2_	-	18	82	-	17	83	40	1	2.5
2-2		-	96	4	-	83	17	40	5	0.05
3-1	SEC_0.4_-PEC-CS_0.8_/PAI-SO_2_	-	20	80	-	18	82	40	1.2	1.84
3-2		-	96	4	-	18	82	51	108	0.008
4-1	CS_0.8_-PEC-SEC_0.4_/PAI-SO_2_	20	-	80	19	-	81	40	1	3.9
4-2		80	-	20	3	-	97	40	127	0.8
4-3		100	-	-	100	-	-	40	-	0.01
5-1	SEC_1.0_-PEC-CS_0.8_/PAI-SO_2_	-	30	70	-	26	74	40	1.2	7.41
5-2		-	80	20	-	2	98	40	181	0.08
5-3		-	96	4	-	14	86	51	150	0.003
6-1	CS_0.8_-PEC-SEC_1.0_/PAI-SO_2_	80	-	20	2	-	98	40	188	0.7
6-2		100	-	-	100	-	-	40	-	0.007
6-3		-	20	80	-	12	88	40	1.8	1.82
6-4		-	80	20	-	2	98	40	190	0.16
6-5		-	96	4	-	65	35	40	13	0.23

*S_F_^w^*, water/alcohol separation factor; *J*, flux value.
